# Association between moderate-to-vigorous physical activity and chronic disease risk in adults and elderly: insights from the UK Biobank study

**DOI:** 10.3389/fphys.2024.1465168

**Published:** 2024-12-05

**Authors:** Kei Shing Ng, Jie Lian, Fan Huang, Yan Yu, Varut Vardhanabhuti

**Affiliations:** ^1^ Snowhill Science Limited, Hong Kong, Hong Kong SAR, China; ^2^ Department of Diagnostic Radiology, The University of Hong Kong, Hong Kong, Hong Kong SAR, China

**Keywords:** physical activity, activity tracker, chronic disease, moderate-to-vigorous physical activity (MVPA), accelerometry, prospective cohort study

## Abstract

**Background:**

This study aimed to determine the associations between different intensities of moderate to vigorous physical activity (MVPA) and the incidence of chronic diseases, and to assess the risk levels associated with these activities over time.

**Methods:**

A prospective cohort study (UK Biobank Activity Project) with data collected between June 2013 and December 2015 included 59,896 adults (mean age = 59.68; male = 38.03%) free from chronic diseases. Participants were categorized into tertiles based on their weekly MVPA: lowest (<224 min for males, <143 min for females), medium (224–444 min for males, 143–308 min for females), and highest (≥444 min for males, ≥308 min for females), stratified by gender. The mean onset of chronic disease symptoms occurred at 3.57 years, with participants followed up during this period. Wearable accelerometry data were used to quantify MVPA levels.

**Findings:**

Lowest tertile of MVPA were significantly correlated with increased risks of chronic disease (24%–110% increased risk) based on odds ratios (ORs), with dose-response relationship observed. In males with the lowest tertile of MVPA, significant associations were identified with type 2 diabetes mellitus (T2DM) (OR = 1.90; CI: 1.44–2.51), neurodegenerative disease (OR = 1.80; CI: 1.19–2.71), metabolic syndrome (OR = 1.34; CI: 1.18–1.53), hypertension (OR = 1.27; CI: 1.12–1.44), and atherosclerotic cardiovascular disease (ASCVD) (OR = 1.24; CI: 1.03–1.49). In females, the lowest tertile of MVPA levels were associated with increased risks of neurodegenerative disease (OR = 2.10; CI: 1.36–3.24), T2DM (OR = 1.88; CI: 1.37–2.58), cerebrovascular disease (OR = 1.61; CI: 1.12–2.29), ASCVD (OR = 1.58; CI: 1.23–2.03), metabolic syndrome (OR = 1.49; CI: 1.32–1.69), and hypertension (OR = 1.44; CI: 1.29–1.61). Longitudinally, the lowest tertile of MVPA in males showed elevated risks for neurodegenerative disease (HR = 2.13; CI: 1.24–3.66), T2DM (HR = 1.83; CI: 1.30–2.57), hypertension (HR = 1.33; CI: 1.15–1.53), metabolic syndrome (HR = 1.32; CI: 1.14–1.54), and ASCVD (HR = 1.29; CI: 1.03–1.61). In females, the lowest tertile of MVPA was associated with similar risks for ASCVD (HR = 1.59; CI: 1.16–2.20), T2DM (HR = 1.57; CI: 1.08–2.29), hypertension (HR = 1.53; CI: 1.34–1.74), and metabolic syndrome (HR = 1.50; CI: 1.29–1.73).

**Conclusion:**

Using wearable accelerometry data, this study demonstrated the quantifiable risks of chronic diseases and their development, highlighting the importance of MVPA.

## 1 Introduction

The prevalence of chronic diseases globally underscores a critical public health challenge, necessitating multifaceted prevention strategies. Among these, physical activity emerges as a modifiable behaviour that can potentially mitigate the risk of numerous non-communicable diseases, including cardiovascular disease, type 2 diabetes, and neurodegenerative disorders (Luo et al., 2023; Dempsey et al., 2022; Alosco et al., 2014; Piercy et al., 2018; Pedersen and Saltin, 2015; Dempsey et al., 2020; Kerr and Booth, 2022). Previous studies have consistently demonstrated a dose-response relationship between physical activity levels and reduced disease risk (Zisko et al., 2017; Tucker et al., 2016), and the use of accelerometers has become increasingly common in research to objectively measure physical activity (Khurshid et al., 2023; Zhong et al., 2023). However, there remains a need for further research integrating objective physical activity data with sociodemographic factors to inform precision exercise prescription strategies aimed at high-risk populations. It is important to distinguish between physical activity and physical exercise, as the two terms refer to different concepts. Physical activity encompasses any bodily movement that increases energy expenditure, such as walking, climbing stairs, or household chores. In contrast, physical exercise refers to structured, planned, and repetitive activities aimed at improving or maintaining physical fitness, such as running or strength training (Dasso, 2019). While this study focuses on physical activity, the insights gained can also inform the precise prescription of physical exercise to mitigate chronic disease risks through personalized interventions. The physiological mechanisms through which moderate-to-vigorous physical activity (MVPA) exerts protective effects against chronic diseases are well-established. Regular MVPA enhances cardiovascular health by improving endothelial function, reducing blood pressure, and promoting favourable lipid profiles (Joyner and Green, 2009). Additionally, MVPA increases insulin sensitivity and glucose uptake, which lowers the risk of developing type 2 diabetes (Colberg et al., 2010). Furthermore, physical activity has been shown to reduce systemic inflammation and oxidative stress, both of which are critical factors in the development of neurodegenerative diseases and metabolic syndrome (Handschin and Spiegelman, 2008). These physiological effects provide a strong rationale for our hypothesis, and we reasoned that higher tertile of MVPA are associated with a reduced risk of chronic disease. The World Health Organization (WHO) has long advocated for regular physical activity as a cornerstone for chronic disease prevention, highlighting its role in reducing the incidence of cardiovascular diseases, diabetes, cancer, and mental health conditions among others (Bull et al., 2020). Despite these recommendations, a significant portion of the global population fails to meet the advised levels of physical activity, contributing to an increased burden of chronic diseases (Doherty et al., 2017).

Recent advances in wearable technology have facilitated more accurate and continuous monitoring of physical activity levels, offering novel insights into the relationship between activity patterns and health outcomes (Gao et al., 2021). Although the WHO (Piercy et al., 2018) recommends that adults aged between 18 and 64 years should accumulate at least 150 min of moderate-intensity aerobic physical activity throughout the week, many believe that the World Health Organization’s (WHO) recommendation of 150 min of moderate physical activity (MPA) per week is no longer sufficient for optimal prevention of chronic diseases, such as cardiovascular disease, type 2 diabetes, and neurodegenerative diseases. Growing evidence suggests that higher tertiles of physical activity, especially at more intense levels, may provide additional benefits for reducing the risk of these conditions. A more detailed and personalized approach to physical activity recommendations is therefore necessary to address the diverse health needs of different populations. (Bull et al., 2020; González et al., 2017; Kyu et al., 2016; Ekelund et al., 2019; Dempsey et al., 2020). The UK Biobank, a large-scale prospective cohort study, has been utilized in numerous studies to assess the relationship between physical activity and health outcomes. However, our study is novel in its integration of objective physical activity measurements from wearable accelerometry with sociodemographic factors such as BMI, age, and income to inform personalized exercise interventions aimed at chronic disease prevention. This approach allows for a potentially more accurate and individualised exploration of how different intensities of physical activity, particularly moderate-to-vigorous physical activity (MVPA), correlate with the risk of developing chronic conditions.

The current study seeks to expand on this body of research by examining the associations between MVPA and the incidence of key chronic diseases within the UK Biobank cohort. We aim to investigate the impact of MVPA on the onset of diseases such as hypertension, type 2 diabetes mellitus, cardiovascular conditions, neurodegenerative diseases (e.g., dementia and Parkinson’s disease), atherosclerotic cardiovascular disease, and metabolic syndrome.

Inherent to the study design, we investigated the dose-response relationship between MVPA and chronic disease risk. Furthermore, by incorporating a wide array of covariates, including demographic, lifestyle, and genetic factors, we attempt to present a holistic analysis that accounts for the multifactorial nature of chronic diseases. Ultimately, our findings aim to emphasise the importance of physical activity in sustaining health and mitigating disease risk.

For establishing relationships between activity tracking data and chronic diseases, we hypothesised that 1) engaging in the lowest or medium tertile of moderate-to-vigorous physical activity (MVPA) is associated with an increased risk of developing chronic diseases among the UK Biobank participants compared to those in the highest tertile of MVPA and 2) the lowest and medium tertile of MVPA are predictive of an increased risk of subsequent development of chronic diseases over 5 years upon follow-up.

## 2 Materials and methods

### 2.1 Ethical approval

This study used data from participants who were part of “The UK Biobank Activity Project”. Prior to participation, written informed consent was obtained to gather questionnaires and biological data. Ethical approval for conducting the UK Biobank study was granted by the UK North West Multi-Centre Research Ethics Committee under reference 11/NW/0382. Written informed consent was secured from all individuals participating in the study. This research has been conducted using the UK Biobank Resource under Application Number 78730. Additionally, this study was approved by the authors’ own local ethics board (UW-20814) at the University of Hong Kong. This study is reported as per STROBE guidelines (von Elm et al., 2007). None of the authors had direct contact with the study participants.

### 2.2 Study population

The UK Biobank is a prospective, population-based cohort study that enrolled over 500,000 volunteers aged 40–69 years between 2006 and 2010 (Sudlow et al., 2015; Zeng et al., 2023). The baseline recruitment targeted participants aged 40–69 years, allowing for the study of middle-aged and older adults, a population critical for understanding age-related health conditions. Initially, a total of 103,669 participants were included in this study who were part of “The UK Biobank Activity Project”, with the data collected between June 2013 and December 2015. To ensure data quality and consistency, we applied several exclusion criteria, including insufficient wear time of the accelerometer, discrepancies between genetic and self-reported sex, pregnancy, and missing key demographic data. Participants with pre-existing chronic diseases, such as hypertension and Type 2 diabetes, were also excluded. After these exclusions, the final study cohort consisted of 59,896 participants.

This sample was then stratified into tertiles based on weekly moderate-to-vigorous physical activity (MVPA) levels, with separate tertiles created for males and females due to differences in average MVPA times. A detailed description of the subject selection process, demographic characteristics, and data collection methods can be found in Sections 2.3–2.5.

The participants were required to wear wrist-worn accelerometers continuously for seven consecutive days using the Axivity AX3 device, which has been validated in previous studies for accurately measuring physical activity levels (Godinho et al., 2016; De Craemer and Verbestel, 2022; Rezende et al., 2024). This protocol was part of the UK Biobank’s standardized approach for objectively assessing physical activity. The use of this device in a large-scale cohort study like the UK Biobank provides robust, reliable and reproducible data on physical activity patterns. This device is a triaxial accelerometer, measuring acceleration in vertical, horizontal, and mediolateral planes. The device is configured to collect tri-axial acceleration data over a seven-day period at a frequency of 100 Hz and a dynamic range of ±8 g.

The follow-up periods varied among participants, but the outcomes relevant to this analysis include the onset of chronic diseases such as hypertension, Type 2 diabetes, cardiovascular conditions, neurodegenerative diseases, and metabolic syndrome. The analysis focused on calculating chronic disease risk over a standardized 5-year period to provide a consistent measure of long-term health outcomes.

### 2.3 Subject selection

**FIGURE 1:**
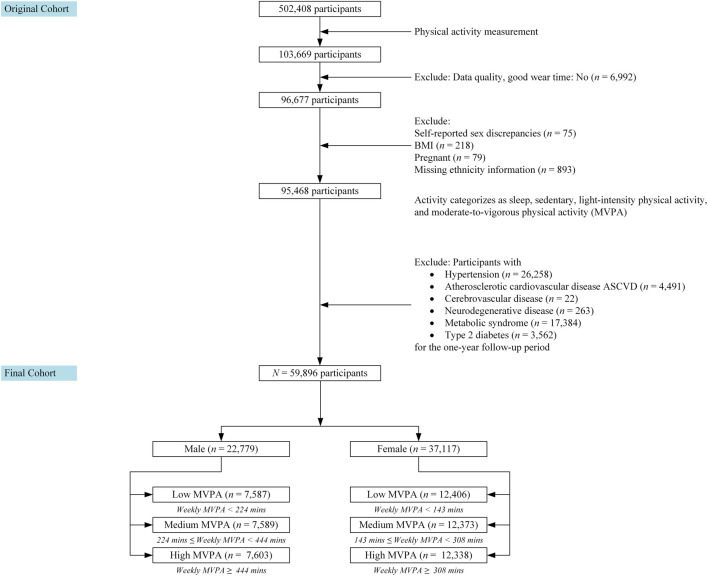
The Study Design and Participants information.

For detailed subject selection, please refer to Figure 1. An initial data preprocessing step was carried out, resulting in the exclusion of participants with insufficient wear time (at least 3 days (72 h) of data and also having data in each 1 h of the 24-h cycle (scattered over multiple days)). As a result, 6,992 participants were excluded from the dataset. Individuals with genetic and self-reported sex discrepancies (*n* = 75), those who were pregnant at baseline (*n =* 79), those with missing BMI data (*n =* 218), and those with missing ethnicity information (*n =* 893) were excluded.

We also excluded participants with pre-existing chronic diseases, including those with Hypertension (*n =* 26,258), Atherosclerotic cardiovascular disease (*n =* 4,491), Cerebrovascular disease (*n =* 22), Neurodegenerative disease (includes Dementia or Parkinson) (*n =* 263), Metabolic Syndrome (*n =* 17,384), and Type 2 diabetes (*n =* 3,562) at the time of accelerometer study or within one-year follow-up period. These exclusions were implemented before incorporating the activity data into the subsequent analysis.

After applying the exclusion criteria, the final study cohort consisted of 59,896 participants. These participants were then stratified into tertiles based on their weekly moderate-to-vigorous physical activity (MVPA) levels. Because the average MVPA times differed between males and females, we created separate tertiles for each gender.

### 2.4 Demographic characteristics

The age range of the participants considered in the analysis ranged from 40 to 69 years old.

### 2.5 Data collection and physical activity classification

Participants were instructed to wear wrist-worn accelerometers continuously for 7 days as part of their involvement in “The UK Biobank Activity Project.” The collected activity data was subjected to classification using a machine-learning pipeline (Supplementary Figure S1) (Doherty et al., 2017). Initially, data processing with calibration against local gravity to standardise outputs across devices to ensure consistency in a large-scale study. Following calibration, periods of non-wear were identified and excluded based on the standard deviation of acceleration signals. The accelerometer data were then subjected to denoising using a fourth-order Butterworth low-pass filter to remove high-frequency noise and improve signal clarity. For activity classification, the cleaned data were segmented into epochs of 5 s, and vector magnitude was calculated to assess the intensity of physical activity. These magnitudes were then used to categorise physical activities into different tertiles based on predetermined thresholds. The categories included sleep, sedentary, light-intensity physical activity, and moderate-to-vigorous physical activity (MVPA). The pipeline focused on threshold-based classification to handle the extensive dataset, facilitating the analysis of physical activity patterns across diverse demographic groups within the cohort. The World Health Organization (WHO) originally recommended a range of 150–300 min of weekly physical activity (Bull et al., 2020). However, based on the actual data, we classified participants into terties based on their levels of physical activity. Individuals were categorised based on their weekly minutes of moderate to vigorous physical activity (MVPA) separately for males and females.

### 2.6 Covariates: demographic and health characteristics

In addition to the accelerometer data, we extracted various participant attributes for incorporation as covariates in our analysis. We included the following factors: age at baseline, body mass index (BMI), ethnic background, income levels, Townsend deprivation index, smoking history, alcohol consumption, measures of social isolation, feelings of loneliness, depression status, dietary habits, medication use, and APOE e4 allele status (for details, please refer to Supplement 1 in Supplementary Material).

### 2.7 Outcomes measures

In this paper, we investigate the association between different tertiles of MVPA levels and the subsequent incidence of chronic diseases among participants.

For the purpose of this study, the chronic diseases refer to Hypertension (HTN), Atherosclerotic Cardiovascular disease (ASCVD), Cerebrovascular disease (CVD), Neurodegenerative disease (ND), Metabolic Syndrome (MetS), Type 2 Diabetes Mellitus (T2DM) and shall be referred herein as “chronic diseases” for ease of reading. As aforementioned, participants with pre-existing chronic diseases were excluded *a priori*.

Subsequent development of these conditions was followed up over a period of up to 5 years, with the last follow-up dated 6 April 2024 (mean follow-up = 3.57 years).

Codes of International Classification of Diseases (ICD) for the chronic diseases are listed in details in Supplement 2 in Supplementary Material.

Incident HTN, ASCVD, CVD, ND, MetS and T2DM were defined as the initial occurrences of respective events observed during follow-up periods with median durations of 3.47 years (interquartile range: 1.79–5.08 years) for HTN, 3.56 years (interquartile range: 1.81–5.19 years) for ASCVD, 3.95 years (interquartile range: 2.25–5.53 years) for CVD, 4.69 years (interquartile range: 3.11–5.84 years) for ND, 3.65 years (interquartile range: 1.98–5.17 years) for MetS, and 3.29 years (interquartile range: 1.64–4.96 years) for T2DM. These definitions resulted in 4,060 incident HTN cases, 1,185 incident ASCVD cases, 393 incident CVD cases, 321 incident ND cases, 3,464 incident MetS cases, and 680 incident T2DM cases.

### 2.8 Statistical analysis

Summary statistics at baseline are reported as percentages for categorical data and as means (and standard deviations) for continuous variables. The odds ratio (OR) (Morris and Gardner, 1988) and its confidence interval (CI) were calculated in order to quantify the strength and direction of the association between different tertiles of MVPA and the risk of developing chronic diseases (for more details, please refer to Supplement 3 in Supplementary Material).

We explored the associations between tertiles of moderate-to-vigorous physical activity (MVPA) and gender, and their respective links to the risk of developing chronic diseases. Our reference group was established as the highest tertile of MVPA group, where the odds ratio was anchored at 1. We examined the odds ratios (ORs) for both the medium and the lowest tertile of MVPA groups in relation to chronic diseases across different genders, which are presented in Table 2 and reported in Section 3.1. The associations of other factors, such as age, BMI, and ethnicity, can be found in Supplementary Table S1 in the Supplementary Material.

For age-specific associations, we designated the age group of <45 years as the reference point. For Body Mass Index (BMI), we designated <25 as the reference group and determined how overweight individuals (BMI = 25–30) and those with obesity (BMI ≥30) related to the odds ratios.

The ethnicity-specific associations were examined by adopting British Caucasians as the reference group. This allowed us to calculate the odds ratios for individuals of Asian ethnicity and individuals from other ethnic backgrounds.

A Multivariable Cox proportional hazards regression model (Andersen and Gill, 1982; Lin and Zelterman, 2002) was applied to estimate the hazard ratios for chronic diseases associated with different tertiles of MVPA over the specified time intervals (3 and 5 years).

We used R (version 4.4.0) in all statistical analyses. A p-value of less than 0.05 was considered statistically significant.

## 3 Results

At baseline, the participants had an average age of 59.68 years (standard deviation: 7.87), with 37,117 individuals (61.97%) identifying as female and 22,779 individuals (38.03%) as male. The majority of the participants, specifically 58,449 individuals (97.58%), self-identified as British Caucasians.

The average MVPA for males is 382.37 min per week (standard deviation: 279.87) and for females is 265.70 min per week (standard deviation: 216.47) in our cohort. The cutoff of different tertiles for males and females were separately calculated being 224 and 444 min per week for males and 143 and 308 min per week for females. This method ensures that the cohort is divided into roughly equal-sized groups based on their levels of physical activity in both gender groups (Refer to Table 1 for baseline characteristics and tertile distribution).

**TABLE 1:** Characteristics of participants by MVPA level and chronic diseases (exclusion of chronic diseases, 12 months cutoff, *N* = 59,896).

Activity level	Male				Female			
	Lowest tertile of MVPA <224 min	Medium tertile of MVPA 224–444 min	Highest tertile of MVPA ≥444 min		Lowest tertile of MVPA <143 min	Medium tertile of MVPA 143–308 min	Highest tertile of MVPA ≥308 min	
*N*	7,587	7,589	7,603		12,406	12,373	12,338	
*%*	33.31	33.32	33.38		33.42	33.34	33.24	
**Covariates**				**p-value**				**p-value**
Age								
<45	92	106	92	ns	113	131	133	ns
≥45 to <60	3,142	3,291	3,365	<0.01	5,305	5,551	6,089	<0.001
≥60	4,353	4,192	4,146	<0.05	6,988	6,691	6,116	<0.001
Mean (SD)	60.37 (8.23)	59.84 (8.08)	59.62 (7.88)	<0.001	60.23 (7.87)	59.64 (7.76)	58.70 (7.50)	<0.001
BMI								
<25.0	2,256	2,897	3,384	<0.001	5,198	6,681	7,888	<0.001
≥25.0 to <30.0	3,817	3,734	3,506	<0.001	4,578	4,248	3,577	<0.001
≥30.0	1,514	958	713	<0.001	2,630	1,444	873	<0.001
Mean (SD)	27.17 (3.86)	26.24 (3.43)	25.75 (3.21)	<0.001	26.77 (4.86)	25.30 (4.01)	24.37 (3.63)	<0.001
Ethnicity								
Asian	137	97	65	<0.001	172	154	110	<0.001
British Caucasian	7,350	7,419	7,472	ns	12,075	12,046	12,087	ns
Other	100	73	66	<0.01	159	173	141	ns
Income levels								
Level 1 (<£18,000)	808	609	604	<0.001	1,770	1,426	1,276	<0.001
Level 2 (£18,000–30,999)	1,557	1,385	1,327	<0.001	2,749	2,598	2,349	<0.001
Level 3 (£31,000–51,999)	2,207	2,061	2,149	ns	3,198	3,174	3,082	ns
Level 4 (≥£52,000)	2,454	3,053	3,096	<0.001	3,072	3,743	4,326	<0.001
Townsend deprivation index quartile								
Q1 (least deprived)	1,982	1,953	1,907	ns	3,207	3,090	2,809	<0.001
Q2	1,879	1,943	1,935	ns	3,251	3,066	2,925	<0.001
Q3	1,949	1,836	1,872	ns	3,065	3,078	3,171	ns
Q4 (most deprived)	1,769	1,852	1,878	ns	2,862	3,123	3,426	<0.001
Smoking status								
Never	4,159	4,415	4,471	<0.001	7,442	7,810	7,780	<0.01
Previous	2,605	2,571	2,624	ns	3,922	3,871	3,988	ns
Current	809	581	501	<0.001	1,023	670	549	<0.001
Alcohol intake								
Daily or almost daily	1,884	2,063	2,143	<0.001	2,132	2,347	2,598	<0.001
3–4 times a week	2,038	2,247	2,403	<0.001	2,712	3,142	3,539	<0.001
1–2 times a week	2,006	1,942	1,886	ns	3,254	3,277	3,148	ns
One to three times a month	748	660	539	<0.001	1,766	1,580	1,366	<0.001
Occasionally	496	402	351	<0.001	1,696	1,354	1,048	<0.001
Never	412	275	280	<0.001	843	669	636	<0.001
Social isolation								
Yes	109	106	116	ns	95	85	78	ns
No	7,415	7,419	7,433	ns	12,203	12,178	12,189	ns
Loneliness								
Yes	971	855	837	<0.001	2,381	2,073	1,917	<0.001
No	6,525	6,652	6,673	ns	9,847	10,156	10,274	<0.01
Depression								
Yes	1,305	1,299	1,225	ns	2,793	2,702	2,642	<0.05
No	1,490	1,520	1,513	ns	1,724	1,875	2,045	<0.001
Diet								
Mean (SD)	12.64 (2.32)	13.03 (2.19)	13.24 (2.16)	<0.001	13.44 (2.08)	13.80 (1.98)	13.98 (1.91)	<0.001
Medication								
Antihypertensive	684	702	738	ns	1,186	1,187	1,156	ns
Blood-glucose lowering	29	31	22	ns	52	43	53	ns
Cholesterol-lowering	666	692	697	ns	1,081	1,124	1,102	ns
APOE								
0	7,468	7,433	7,483	ns	12,154	12,142	12,112	ns
1	0	0	0	ns	0	0	0	ns
2	119	156	120	ns	252	231	226	ns
Chronic diseases								
Hypertension	720	603	498	<0.001	1,012	653	574	<0.001
Atherosclerotic cardiovascular disease	298	241	217	<0.001	195	133	101	<0.001
Cerebrovascular disease	68	74	45	<0.05	89	66	51	<0.01
Neurodegenerative disease	69	37	56	ns	57	71	31	<0.05
Metabolic syndrome	658	495	452	<0.001	769	630	460	<0.001
T2 Diabetes	197	102	72	<0.001	168	87	54	<0.001

Diet: A composite score, ranging from 0 to 20, was calculated based on the frequency of consumption of vegetables, fruits, fish, and processed and red meat (inverted), with a higher score indicating a more favourable dietary pattern, excluding preferences for red meat. The scoring system was as follows: Never = 0, Less than once a week = 1, Once a week = 2, 2–4 times a week = 3, 4–6 times a week = 4, Once or more daily = 5. Notably, the score for processed and red meat intake was reversed in the calculation.

Data are *n* or *n* (%) or mean (sd).

The mean (SD) MVPA in the lowest tertile was 124.98 (61.75) minutes per week for males and 72.72 (41.56) minutes per week for females. In the medium tertile, the mean (SD) MVPA was 326.93 (62.62) minutes per week for males and 219.56 (46.91) minutes per week for females. In the highest tertile, the mean (SD) MVPA was 694.25 (245.99) minutes per week for males and 506.03 (199.20) minutes per week for females.

### 3.1 Activity level and association with chronic diseases

For both genders, lowest tertiles of moderate-to-vigorous physical activity (MVPA) were associated with a higher risk of hypertension, atherosclerotic cardiovascular disease, neurodegenerative disease, metabolic syndrome, and type 2 diabetes compared to medium tertiles of MVPA (see Table 2 for odds ratios).

**TABLE 2:** Odds ratios, 95% confidence intervals, and p-values for chronic diseases by activity levels and gender.

	Hypertension	Atherosclerotic cardiovascular disease	Cerebrovascular disease	Neurodegenerative disease	Metabolic syndrome	Type 2 diabetes
Male, n	1,821	756	187	162	1,605	371
Activity Level						
Highest tertile of MVPA	1	1	1	1	1	1
Medium tertile of MVPA	1.18 (1.04, 1.33) p < 0.05	1.08 (0.89, 1.30) p = ns	1.66 (1.14, 2.41) p < 0.01	1.54 (1.02, 2.35) p < 0.05	1.06 (0.93, 1.22) p = ns	1.26 (0.92, 1.70) p = ns
Lowest tertile of MVPA	1.27 (1.12, 1.44) p < 0.001	1.24 (1.03, 1.49) p < 0.05	1.43 (0.97, 2.11) p = ns	1.80 (1.19, 2.71) p < 0.01	1.34 (1.18, 1.53) p < 0.001	1.90 (1.44, 2.51) p < 0.001
Female, n	2,239	429	206	159	1,859	309
Activity Level						
Highest tertile of MVPA	1	1	1	1	1	1
Medium tertile of MVPA	1.03 (0.91, 1.15) p = ns	1.20 (0.93, 1.57) p = ns	1.22 (0.85, 1.77) p = ns	1.71 (1.10, 2.66) p < 0.05	1.30 (1.14, 1.47) p < 0.001	1.28 (0.91, 1.81) p = ns
Lowest tertile of MVPA	1.44 (1.29, 1.61) p < 0.001	1.58 (1.23, 2.03) p < 0.001	1.61 (1.12, 2.29) p < 0.001	2.10 (1.36, 3.24) p < 0.001	1.49 (1.32, 1.69) p < 0.001	1.88 (1.37, 2.58) p < 0.001

Male participants with lowest tertile of MVPA in general exhibited a higher risk factor for chronic diseases than those with medium tertile of MVPA. The highest ORs were observed for Type 2 Diabetes with an OR of 1.90 (95% CI: 1.44 to 2.51, p < 0.001), followed by neurodegenerative disease with an OR of 1.80 (95% CI: 1.19 to 2.71, p < 0.01), metabolic syndrome with an OR of 1.34 (95% CI: 1.18 to 1.53, p < 0.001), hypertension with an OR of 1.27 (95% CI: 1.12 to 1.44, p < 0.001) and atherosclerotic cardiovascular disease exhibited an OR of 1.24 (95% CI: 1.03 to 1.49, p < 0.05). However, Cerebrovascular disease showed a non-significant association, OR of 1.43 (95% CI: 0.97 to 2.11, p = ns).

For male participants engaging in medium tertile of MVPA, varying degrees of risk associated with chronic diseases were observed compared to the reference group. The highest odds ratio (OR) was observed in Cerebrovascular diseases with an OR of 1.66 (95% CI: 1.14 to 2.41, p < 0.01), followed by neurodegenerative disease, with an OR of 1.54 (95% CI: 1.02 to 2.35, p < 0.05), hypertension with an OR of 1.18 (95% CI: 1.04 to 1.33, p < 0.05). However, for T2 Diabetes, ASCVD and metabolic syndrome displayed non-statistically significant ORs of 1.26 (95% CI: 0.92 to 1.70, p = ns), 1.08 (95% CI: 0.89 to 1.30, p = ns) and 1.06 (95% CI: 0.93 to 1.22, p = ns), respectively.

For female individuals with lowest tertile of MVPA in general exhibited a higher risk factor for chronic diseases than those with medium tertile of MVPA. Neurodegenerative disease showed the highest OR at 2.10 (95% CI: 1.36 to 3.24, p < 0.001), followed by Type 2 Diabetes with an OR of 1.88 (95% CI: 1.37 to 2.58, p < 0.001), Cerebrovascular diseases with an OR of 1.61 (95% CI: 1.12 to 2.29, p < 0.001), ASCVD with an OR of 1.58 (95% CI: 1.23 to 2.03, p < 0.001), Metabolic syndrome with an OR of 1.49 (95% CI: 1.32 to 1.69, p < 0.001), and Hypertension with an OR of 1.44 (95% CI: 1.29 to 1.61, p < 0.001).

For female participants engaging in medium tertile of MVPA, the highest odds ratio (OR) was observed for neurodegenerative disease with an OR of 1.71 (95% CI: 1.10 to 2.66, p < 0.05) followed by Metabolic syndrome with an OR of 1.30 (95% CI: 1.14 to 1.47, p < 0.001). However, T2 Diabetes, Cerebrovascular diseases, ASCVD and Hypertension displayed ORs of 1.28 (95% CI: 0.91 to 1.81, p = ns), 1.22 (95% CI: 0.85 to 1.77, p = ns), 1.20 (95% CI: 0.93 to 1.57, p = ns) and 1.03 (95% CI: 0.91 to 1.15, p = ns) respectively, indicating a non-significant association.

Except for cerebrovascular disease in males, there is a dose-response relationship for all groups of chronic diseases, in that subjects with lowest tertile of MVPA have a higher risk than the medium tertile of MVPA who also have a higher risk than the highest tertile of MVPA groups.

The proportions of participants with chronic diseases within each tertile of MVPA were as follows: in the lowest tertile, 21.73% of participants had a chronic disease by the time of data collection, compared to 16.06% in the medium tertile and 13.02% in the highest tertile. These results suggest a dose-response relationship, with the highest tertile of MVPA associated with lower overall chronic disease prevalence.

### 3.2 Prediction of subsequent development of chronic diseases

**FIGURE 2:**
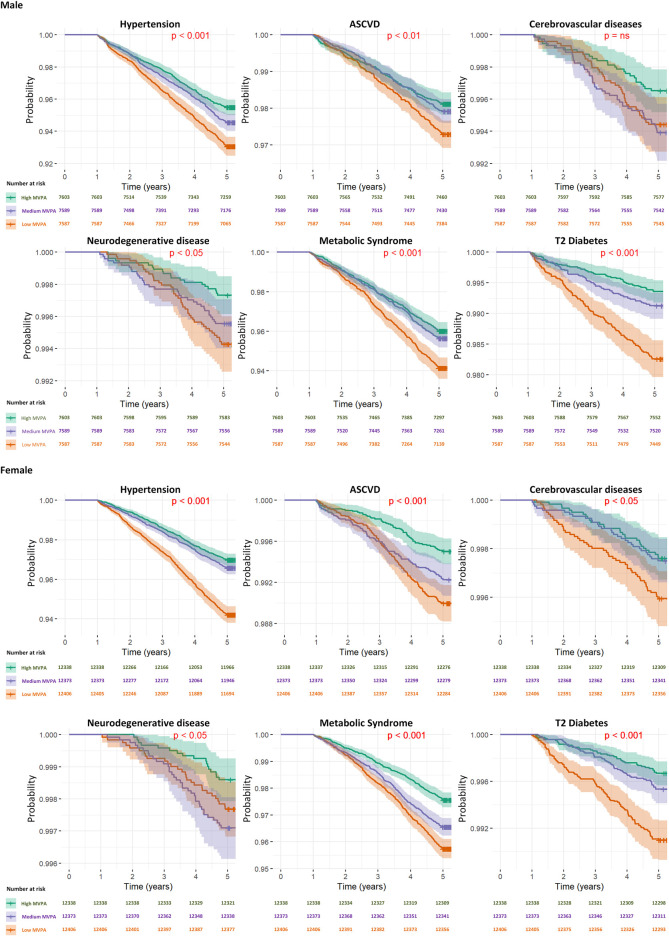
Kaplan–Meier survival curves for diseases specific mortality across activity levels by gender.

We conducted a comprehensive analysis of the relationship between moderate-to-vigorous physical activity (MVPA) levels, gender, age, BMI, and the development of chronic diseases over varying periods of 3 and 5 years. We chose to primarily focus on the 5-year follow-up data as it provides a more comprehensive assessment of the long-term impact of MVPA on the development of chronic diseases. Chronic conditions, such as cardiovascular and neurodegenerative diseases, typically take longer to manifest, and the extended 5-year period allows for a more accurate evaluation of these associations. Shorter follow-up periods may not fully capture the progression of such conditions. The results for the 3-year timeframe can be found in Supplementary Table S2, and the results for the 5-year timeframe are presented in Table 3 and Figure 2. Cox regression model considered several covariates, including age at baseline, BMI, ethnic background, income levels, Townsend deprivation index, smoking history, alcohol consumption, hypertension status, measures of social isolation, feelings of loneliness, depression status, dietary habits, medication use, and APOE e4 allele status.

**TABLE 3:** Hazard ratio of chronic disease development at 5 years with MVPA levels, gender, age, and BMI.

	Hypertension		Atherosclerotic cardiovascular disease		Cerebrovascular disease		Neurodegenerative disease		Metabolic syndrome		Type 2 diabetes	
	Model 1	Model 2	Model 1	Model 2	Model 1	Model 2	Model 1	Model 2	Model 1	Model 2	Model 1	Model 2
Total participant												
Highest	HR = 1											
Medium	1.17 (1.06, 1.30)	1.07 (0.97, 1.19)	1.24 (1.03, 1.50)	1.15 (0.95, 1.39)	1.37 (0.97, 1.94)	1.31 (0.93, 1.86)	1.85 (1.24, 2.76)	1.78 (1.19, 2.67)	1.26 (1.13, 1.40)	1.17 (1.05, 1.31)	1.38 (1.05, 1.82)	1.13 (0.86, 1.49)
p-value	<0.01	ns	<0.05	ns	ns	ns	<0.01	<0.01	<0.001	<0.01	<0.05	ns
Lowest	1.76 (1.60, 1.93)	1.42 (1.29, 1.56)	1.62 (1.35, 1.93)	1.34 (1.11, 1.60)	1.65 (1.18, 2.31)	1.49 (1.06, 2.11)	1.91 (1.28, 2.84)	1.78 (1.18, 2.67)	1.62 (1.46, 1.79)	1.39 (1.25, 1.54)	2.72 (2.13, 3.48)	1.67 (1.30, 2.15)
p-value	<0.001	<0.001	<0.001	<0.01	<0.01	<0.05	<0.01	<0.01	<0.001	<0.001	<0.001	<0.0001
Male												
Highest	HR = 1 (reference)											
Medium	1.21 (1.05, 1.40)	1.15 (1.00, 1.33)	1.11 (0.88, 1.39)	1.07 (0.85, 1.34)	1.75 (1.08, 2.83)	1.77 (1.09, 2.87)	1.66 (0.95, 2.90)	1.70 (0.97, 2.97)	1.09 (0.93, 1.28)	1.05 (0.90, 1.23)	1.37 (0.94, 1.98)	1.19 (0.82, 1.73)
p-value	<0.01	ns	ns	ns	<0.05	<0.05	ns	ns	ns	ns	ns	ns
Lowest	1.56 (1.36, 1.79)	1.33 (1.15, 1.53)	1.45 (1.17, 1.80)	1.29 (1.03, 1.61)	1.60 (0.98, 2.62)	1.54 (0.93, 2.55)	2.14 (1.25, 3.64)	2.13 (1.24, 3.66)	1.48 (1.28, 1.72)	1.32 (1.14, 1.54)	2.73 (1.96, 3.80)	1.83 (1.30, 2.57)
p-value	<0.001	<0.001	<0.001	<0.05	ns	ns	<0.01	<0.01	<0.001	<0.001	<0.001	<0.001
Female												
Highest	HR = 1 (reference)											
Medium	1.14 (0.99, 1.31)	1.02 (0.88, 1.17)	1.56 (1.13, 2.16)	1.40 (1.01, 1.94)	1.04 (0.62, 1.73)	0.95 (0.57, 1.59)	2.07 (1.16, 3.69)	1.96 (1.10, 3.51)	1.42 (1.22, 1.65)	1.31 (1.13, 1.52)	1.41 (0.94, 2.11)	1.09 (0.72, 1.64)
p-value	ns	ns	<0.01	<0.05	ns	ns	<0.05	<0.05	<0.001	<0.001	ns	ns
Lowest	1.95 (1.72, 2.21)	1.53 (1.34, 1.74)	2.02 (1.48, 2.76)	1.59 (1.16, 2.20)	1.69 (1.07, 2.68)	1.49 (0.93, 2.40)	1.65 (0.90, 3.01)	1.48 (0.80, 2.76)	1.76 (1.53, 2.03)	1.50 (1.29, 1.73)	2.74 (1.90, 3.93)	1.57 (1.08, 2.29)
p-value	<0.001	<0.001	<0.001	<0.01	<0.05	ns	ns	ns	<0.001	<0.001	<0.001	<0.05
Male Age												
<45	HR = 1 (reference)											
45–60		10.03 (1.41, 71.52)		1.78 (0.44, 7.24)		NA		NA		1.29 (0.53, 3.13)		0.39 (0.16, 0.99)
p-value		<0.05		ns						ns		<0.05
≥60		20.71 (2.91, 147.43)		4.29 (1.06, 17.31)		NA		NA		3.46 (1.43, 8.37)		0.77 (0.31, 1.93)
p-value		<0.01		<0.05						<0.01		ns
Female Age												
<45	HR = 1 (reference)											
45–60		2.46 (0.79, 7.69)		NA		NA		NA		2.05 (0.66, 6.40)		0.53 (0.16, 1.69)
p-value		ns								ns		ns
≥60		5.67 (1.82, 17.64)		NA		NA		NA		5.26 (1.69, 16.41)		0.77 (0.24, 2.48)
p-value		<0.01								<0.01		ns
Male BMI												
<25	HR = 1 (reference)											
25–30		1.36 (1.19, 1.56)		1.15 (0.94, 1.41)		0.60 (0.40, 0.90)		0.79 (0.51, 1.21)		1.04 (0.90, 1.19)		1.65 (1.16, 2.36)
p-value		<0.001		ns		<0.05		ns		ns		<0.01
≥30		2.08 (1.76, 2.44)		1.37 (1.04, 1.79)		0.55 (0.29, 1.02)		0.57 (0.27, 1.19)		1.35 (1.13, 1.62)		4.55 (3.14, 6.58)
p-value		<0.001		<0.05		ns		ns		<0.01		<0.001
Female BMI												
<25	HR = 1 (reference)											
25–30		1.30 (1.16, 1.47)		1.28 (0.98, 1.69)		1.19 (0.79, 1.81)		0.74 (0.45, 1.22)		1.15 (1.01, 1.30)		3.18 (2.13, 4.76)
p-value		<0.001		ns		ns		ns		<0.05		<0.001
≥30		2.27 (1.98, 2.61)		1.87 (1.35, 2.59)		1.05 (0.58, 1.91)		0.87 (0.44, 1.72)		1.37 (1.17, 1.62)		8.50 (5.67, 12.73)
p-value		<0.001		<0.001		ns		ns		<0.01		<0.001

Model 1: non-adjusted, Model 2: adjusted for age, sex, BMI, ethnicity, Income Level, Townsend Deprivation Index, Smoking Status, Alcohol Intake, Social Isolation, Loneliness, Depression, Dietary Score, Medication, and APOE.

*A HR, marked as NA may imply that the calculation cannot be performed due to the hazard rate in the reference group being zero, indicating that no events have occurred in this group. Consequently, the HR, becomes undefined because it relies on comparing event rates between two groups.

The results, presented in Table 3 and Figure 2, provide hazard ratios (HRs) and Kaplan-Meier curve for specific diseases, including Hypertension, Atherosclerotic Cardiovascular disease, Cerebrovascular disease, Neurodegenerative disease, Metabolic Syndrome, and Type 2 Diabetes Mellitus, for 5-year timeframes. These HRs are accompanied by their respective 95% confidence intervals (CIs) and p-values. For results of the development of chronic diseases at the 3-year timeframes, readers are directed to Supplementary Table S2 in Supplementary Material.

### 3.3 Activity level

In our study assessing the impact of tertiles of moderate-to-vigorous physical activity (MVPA) on the development of chronic diseases over 5-year timeframes, significant hazard ratios (HRs) were observed in health outcomes based on activity levels. For ease of reading, we will discuss adjusted HRs at 5 years as follows.

For individuals in the medium tertile of MVPA, the overall risk of developing chronic diseases was generally lower compared to the lowest tertile of MVPA group.

Among individuals categorised within the lowest tertile of MVPA group, we identified increased risks for several chronic conditions. Since there were some differential effects based on gender, notably for neurodegenerative disease, we will discuss the HRs separately by gender. For example, neurodegenerative disease presented the highest hazard ratio (HR = 1.78, 95% CI: 1.18 to 2.67, p < 0.01) for the lowest tertile of MVPA group, indicating a substantial increase in risk, although based on gender sub-analyses, this effect seems only to be confined and driven by the male group.

#### 3.3.1 Male participants

For males categorised within the lowest tertile of MVPA, a significantly increased risk was observed for several conditions. Neurodegenerative disease presented the most substantial risk increase (HR = 2.13, 95% CI: 1.24 to 3.66, p < 0.01), followed by type 2 diabetes (HR = 1.83, 95% CI: 1.30 to 2.57, p < 0.001), hypertension (HR = 1.33, 95% CI: 1.15 to 1.53, p < 0.001), metabolic syndrome (HR = 1.32, 95% CI: 1.14 to 1.54, p < 0.001) and atherosclerotic cardiovascular disease (HR = 1.29, 95% CI: 1.03 to 1.61, p < 0.05). However, cerebrovascular disease did not show a significant risk (HR = 1.54, 95% CI: 0.93 to 2.55, p = ns).

Among males engaging in a medium tertile of MVPA, there was a generally lower risk for chronic diseases compared to those in the lowest tertile of MVPA group, with the exception of cerebrovascular diseases. The observed hazard ratios (HRs) for chronic disease development were as follows: cerebrovascular diseases presented a significant risk increase (HR = 1.77, 95% CI: 1.09 to 2.87, p < 0.05). Conversely, neurodegenerative disease (HR = 1.70, 95% CI: 0.97 to 2.97, p = ns), type 2 diabetes (HR = 1.19, 95% CI: 0.82 to 1.73, p = ns), hypertension (HR = 1.15, 95% CI: 1.00 to 1.33, p = ns), atherosclerotic cardiovascular disease (HR = 1.07, 95% CI: 0.85 to 1.34, p = ns) and metabolic syndrome (HR = 1.05, 95% CI: 0.90 to 1.23, p = ns) did not demonstrate statistically significant risks.

#### 3.3.2 Female participants

Among females in the lowest tertile of MVPA category, significant risks were also identified, with ASCVD showing the highest risk increase (HR = 1.59, 95% CI: 1.16 to 2.20, p < 0.01), followed by T2DM (HR = 1.57, 95% CI: 1.08 to 2.29, p < 0.05), hypertension (HR = 1.53, 95% CI: 1.34 to 1.74, p < 0.001), and metabolic syndrome (HR = 1.50, 95% CI: 1.29 to 1.73, p < 0.001). Cerebrovascular diseases (HR = 1.49, 95% CI: 0.93 to 2.40, p = ns), and neurodegenerative disease (HR = 1.48, 95% CI: 0.80 to 2.76, p = ns) did not exhibit significant risks at the 5-year.

For females with a medium tertile of MVPA, the pattern of chronic disease risk over 5 years was broadly similar to that of males, with neurodegenerative disease showing a significant increase in risk (HR = 1.96, 95% CI: 1.10 to 3.51, p < 0.05), followed by ASCVD (HR = 1.40, 95% CI: 1.01 to 1.94, p < 0.05), metabolic syndrome (HR = 1.31, 95% CI: 1.13 to 1.52, p < 0.001). However, no statistically significant risks were found for T2DM (HR = 1.09, 95% CI: 0.72 to 1.64, p = ns), hypertension (HR = 1.02, 95% CI: 0.88 to 1.17, p = ns), and cerebrovascular diseases (HR = 0.95, 95% CI: 0.57 to 1.59, p = ns).

The analysis stratified by age and BMI can be found in Supplement 4 in Supplementary Material.

## 4 Discussion

Our analysis of the UK Biobank cohort study provides evidence of the protective effects of moderate-to-vigorous physical activity (MVPA) against the development of chronic diseases. While physical activity was measured only at baseline using accelerometry, the longitudinal dimension of this study stems from tracking the development of chronic diseases over time. By examining both Odds Ratios (ORs) and Hazard Ratios (HRs), we reveal the multifaceted role of physical activity in relation to health outcomes across both cross-sectional and longitudinal perspectives.

We observed a gradient with a dose-response relationship across multiple chronic diseases across different levels of MVPA. Participants engaging in lowest tertile of MVPA, who still met the current WHO recommendations of a minimum of 150 min per week, faced significantly higher odds of developing conditions such as hypertension, atherosclerotic cardiovascular disease, neurodegenerative disease, metabolic syndrome, and type 2 diabetes, compared to their counterparts in higher MVPA tertiles (Kyu et al., 2016) These findings align with the WHO’s recommendations on physical activity as a pivotal element in chronic disease prevention. However, it is important to note that some participants in our cohort did not meet the WHO guidelines of 150 min of moderate-to-vigorous physical activity per week. This variation highlights the need for continued public health efforts to ensure more individuals meet or exceed these activity levels to fully benefit from the protective effects against chronic diseases.

Secondly, the Hazard Ratios (HRs) extended these findings over a 5-year follow-up period, offering a longitudinal perspective on the relationship between MVPA levels and chronic disease development. Similar to the ORs, the HRs demonstrated an increased risk of developing chronic diseases, such as hypertension, type 2 diabetes, and cardiovascular conditions, when tracked over a long follow-up time. Particularly notable was the substantial reduction in risk for hypertension and type 2 diabetes among participants with highest tertile of MVPA, even after adjusting for a wide range of covariates.

Our gender-specific sub-analysis showed distinct patterns of association, suggesting differential impacts of physical activity on men and women. That is, lowest tertile of MVPA was associated with an increased risk of neurodegenerative disease in men and elevated risks of cerebrovascular diseases and type 2 diabetes in women. These gender-specific findings underscore the importance of tailored physical activity recommendations stratified by gender.

The stratified analysis by age and BMI categories illustrated the compounded risks faced by older adults and individuals with higher BMI who engage in the lowest tertile of physical activity. The effects of the highest tertile of MVPA across all demographic segments highlight the broad applicability of physical activity as a preventive strategy.

The recorded incidence of 16% of participants developing a chronic disease is consistent with epidemiological data from populations of similar age and gender composition. Research suggests that the incidence of chronic conditions such as cardiovascular disease and type 2 diabetes increases with age, with approximately 20%–25% of individuals in their 60s and 70s experiencing one or more chronic conditions. Thus, the incidence observed in our study aligns with expected rates for this demographic.

The consistent protective effects of moderate-to-vigorous physical activity (MVPA) against a spectrum of chronic diseases underscore the critical importance of promoting physical activity as a key public health strategy. The reduction in chronic disease risk observed in participants with the highest tertiles of MVPA can be explained by several well-documented physiological mechanisms. MVPA promotes cardiovascular health by enhancing endothelial function and reducing arterial stiffness, which in turn lowers the risk of hypertension and cardiovascular diseases (Gao et al., 2022). Additionally, highest tertile of physical activity are associated with improvements in insulin sensitivity and reductions in visceral fat, which directly mitigate the risk of metabolic syndrome and type 2 diabetes (Ibañez et al., 2005). Regular physical activity also reduces systemic inflammation, a key contributor to both neurodegenerative diseases and metabolic syndrome (Dempsey et al., 2020; Chomiuk et al., 2024). These physiological effects highlight how our findings align with the known benefits of physical activity and support the use of precise exercise prescription strategies for chronic disease prevention. Our findings support the well-established association between the highest tertile of MVPA and reduced risk of chronic diseases, as demonstrated in numerous previous studies (Luo et al., 2023; Dempsey et al., 2022; Zisko et al., 2017). However, our study adds to the existing literature by using a large-scale cohort with wearable accelerometry data to objectively measure MVPA, which offers more precision than self-reported methods typically used in earlier studies (Khurshid et al., 2023; Doherty et al., 2017; Wu et al., 2021). Furthermore, by incorporating additional covariates such as BMI, age, and household income, we provide new insights into how these factors modulate both physical activity levels and chronic disease risk over time, paving the way for personalized exercise interventions that can be tailored to individual risk profiles. Although this study primarily examined moderate-to-vigorous physical activity (MVPA) levels in the context of chronic disease prevention, these findings have important implications for the field of precision exercise prescription. Our data-driven approach, utilizing wearable accelerometry, provides a valuable tool for assessing baseline activity levels and developing individualized exercise programs tailored to specific health risks. For example, individuals at higher risk of chronic diseases could benefit from personalized exercise interventions that exceed the general physical activity recommendations. Such targeted programs could optimize health outcomes by accounting for individual differences in fitness levels, baseline activity, and disease susceptibility.

By integrating accelerometry data with individualized health risk profiles, practitioners can develop precise exercise prescriptions that not only meet but also exceed the minimum physical activity guidelines, especially for high-risk populations. This approach aligns with the objectives of precision exercise prescription, which seeks to move beyond generalized recommendations towards more tailored, effective interventions. This imperative has become even more pronounced in the context of the COVID-19 pandemic. As the pandemic ushered in widespread shifts towards remote work and home-based lifestyles, many individuals have experienced significant reductions in daily physical activity. The transition to working from home, while necessary for public health measures, inadvertently fostered environments conducive to more sedentary behaviours due to the proximity of personal living spaces to workstations and the elimination of commute-related physical activity (Gao and Lee, 2022; Stockwell et al., 2021; Lindberg et al., 2023). The decreased engagement in physical activity during the pandemic has potential long-term implications for public health, particularly in the escalation of chronic disease risk factors. Our findings reveal that even small increases in physical activity, while not necessarily meeting the recommended levels, can lead to significant health improvements and a reduced risk of developing chronic diseases. A personalised approach to boost activity levels for various groups, considering differences in gender, age, and weight-related challenges should be adopted.

### 4.1 Practical applicability

The findings of this study have significant practical implications for public health and personalized medicine. The observed associations between moderate-to-vigorous physical activity (MVPA) and the reduced risk of chronic diseases underscore the importance of promoting regular physical activity across middle-aged and older populations. By leveraging wearable accelerometry data, this study provides a scalable and objective method for monitoring physical activity, which can be incorporated into routine healthcare practices. The stratification of participants by activity levels allows for tailored physical activity recommendations, highlighting the potential for interventions that are customized to individual needs based on gender, age, and baseline activity levels. These insights can inform the development of community-based programs and public health campaigns aimed at reducing the burden of chronic diseases through increased physical activity.

There are some limitations worth noting. First, the activity tracker was based on accelerometry data and did not take into account physiological data (e.g., heart rate, respiratory rates, etc.). Our model is an estimation of the minutes involved in moderate to vigorous activity, and there may be situations where this may not reflect true body states. An example of this may be a person on an exercise bike who remains in a stationary position but is engaging in moderate to vigorous activity. Future models, should take into consideration physiological data. It is possible that in some individuals, the MVPA is underestimated, but given the relatively large sample size, we believe the findings are still valid.

Additionally, the study population primarily consisted of British Caucasians between 45 and 60 years old, which may limit the generalizability of the findings to other ethnicities and age groups. Expanding future studies to include younger and older populations, as well as more diverse ethnic groups, would provide a broader understanding of physical activity’s impact across different demographic segments. Moreover, the observational nature of the study limits the ability to establish causality between MVPA and chronic disease outcomes. Although we controlled for a wide range of covariates, unmeasured confounders may still have influenced the results. Future studies should explore longitudinal or randomized controlled designs to establish clearer causal pathways.

Second, owing to the cohorts of the study with the majority of subjects being British Caucasian and age between 45–60 years old, the study may lack broader applicability. Future studies expanding into other age groups as well as other ethnicities may be warranted. Finally, considering our study leveraged data from the UK Biobank, where participants were required to wear activity-tracking accelerometers, the potential for a Hawthorne effect may need to be borne in mind. This observational bias might have led participants to modify their activity levels, possibly increasing their moderate-to-vigorous physical activity (MVPA) due to the awareness of being monitored. Such adjustments could skew the interpretations of physical activity’s impact on chronic disease risk.

It is important to note that within the lowest tertile of MVPA, there are participants who do not meet the recommended guideline of 150 min of MVPA per week, as well as those who do. This variation within the lowest tertile could influence the observed association between MVPA and chronic disease risk. Individuals not meeting the guidelines may have a disproportionately higher risk, potentially driving the association seen in this group. Future studies could stratify participants further based on whether they meet physical activity guidelines to refine these findings.

Building on these limitations, we also recognize that when factors such as BMI, age, and household income are adjusted for, the initially observed relationship between the lowest tertile MVPA and increased risk of chronic diseases diminishes. This indicates that these factors are significant mediators in the relationship between physical activity and chronic disease risk. Older age, higher BMI, and lower income levels may independently contribute to both lower levels of physical activity and increased disease risk. These results highlight the importance of addressing multiple lifestyle and socioeconomic factors when developing public health strategies to reduce chronic disease incidence.

Despite these limitations, the findings emphasize the importance of physical activity interventions tailored to individual risk profiles. Public health strategies could leverage accelerometry data for personalized recommendations, encouraging even small increases in MVPA for meaningful health benefits.

Future research should focus on unravelling the biological mechanisms underlying the protective effects of physical activity, which could inform more targeted prevention and intervention strategies. Exploring effective ways to sustain high levels of physical activity over time remains a crucial area for public health intervention.

### 4.2 Novelty and contribution to the field

While previous studies have established the relationship between physical activity and chronic disease prevention, our study offers several novel contributions. First, we utilized wearable accelerometry to objectively measure physical activity in a large cohort, providing more accurate assessments of MVPA compared to self-reported data, which reduces bias and enhances validity. Second, by integrating sociodemographic factors such as BMI, age, and income, we offer a more comprehensive analysis of how these variables modulate both physical activity levels and chronic disease risk, paving the way for personalized exercise interventions. Finally, our study includes long-term follow-up data, demonstrating the sustained benefits of MVPA over several years, which adds to the understanding of how continuous engagement in physical activity impacts chronic disease risk over time.

## 5 Conclusion

In summary, our study provides evidence that engaging in the highest tertile of MVPA is associated with significant reductions in the risk of developing chronic diseases. We demonstrated quantifiable risks based on in different groups based on easily accessible wearable data. These findings reinforce the value of physical activity as a cornerstone of chronic disease prevention and health promotion, highlighting the need for sustained efforts to increase physical activity levels in the general population.

## Data Availability

The datasets presented in this article are not readily available because researchers must apply to access the UK Biobank dataset. Requests to access the datasets should be directed to https://www.ukbiobank.ac.uk/.
