# Supplementary figures and images for: Association between moderate-to-vigorous physical activity and chronic disease risk in adults and elderly: insights from the UK Biobank study
Source: Front Physiol. 2024 Dec 5;15:1465168. doi: 10.3389/fphys.2024.1465168 (PMC11659758; doi:10.3389/fphys.2024.1465168)

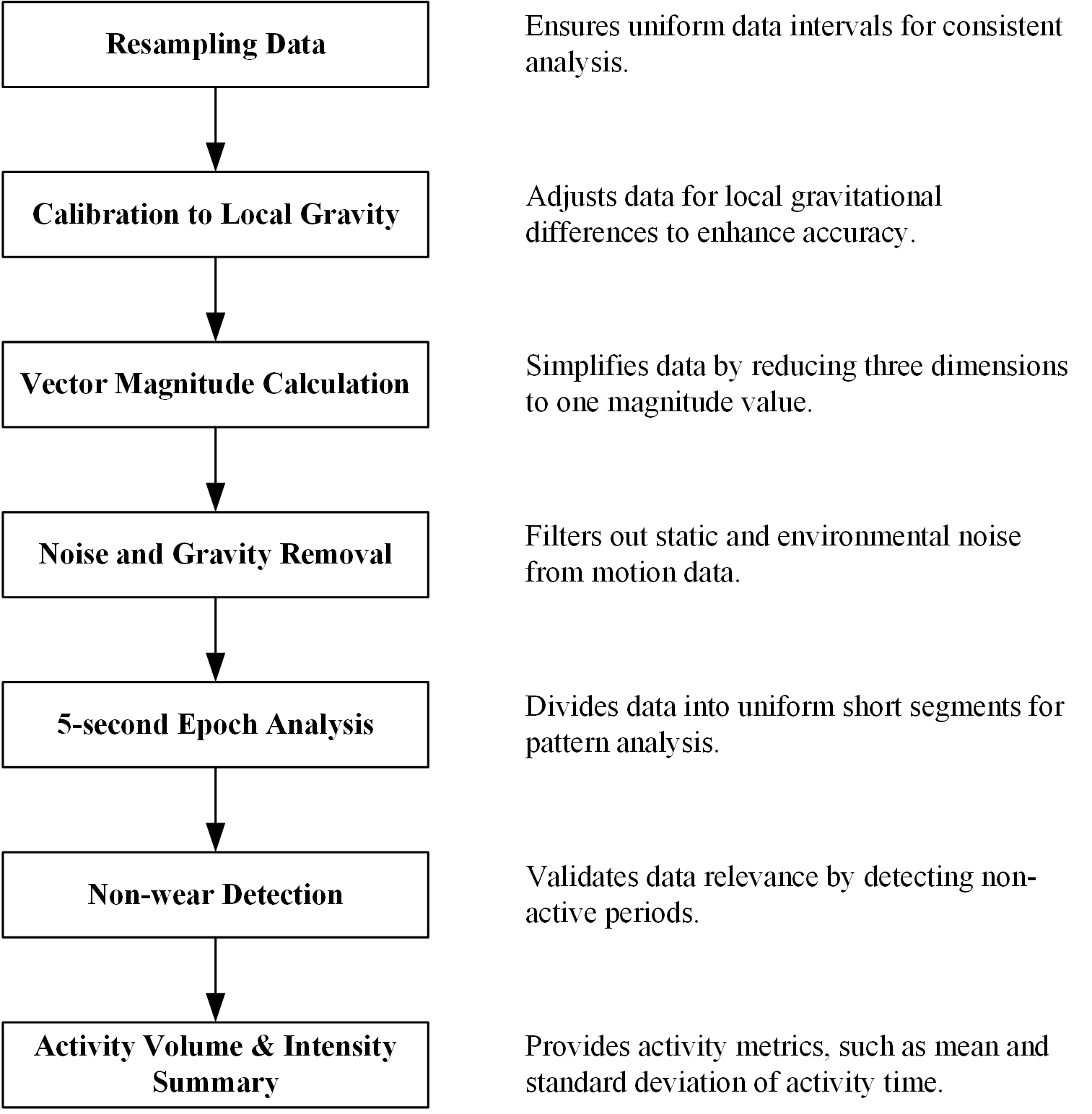

Supplement: Supplementary file 2 [file Image1.jpeg]
