# Supplementary material for: Association between moderate-to-vigorous physical activity and chronic disease risk in adults and elderly: insights from the UK Biobank study
Source: Front Physiol. 2024 Dec 5;15:1465168. doi: 10.3389/fphys.2024.1465168 (PMC11659758; doi:10.3389/fphys.2024.1465168)
Supplement: Supplementary file 3 [file Table2.docx]

**Supplementary 1**

Definition of Covariate

**Body Mass Index (BMI) Categories**:

BMI was calculated as weight in kilograms divided by the square of height in meters (kg/m²). Categories were defined according to the World Health Organization (WHO) criteria:

- Underweight: BMI < 18.5 kg/m²
- Normal weight: BMI 18.5–24.9 kg/m²
- Overweight: BMI 25.0–29.9 kg/m²
- Obesity: BMI ≥ 30 kg/m²

**Ethnicity Classification**:

Participants self-reported their ethnicity, which was categorized into three main groups for analysis:

- British Caucasian
- Asian (including any sub-groups identified by participants)
- Other (encompassing any ethnicities not classified as British Caucasian or Asian)

**Annual Household Income Brackets**:

Income levels were self-reported, with participants selecting the bracket that best described their annual household income before taxes:

- Level 1: <£18,000
- Level 2: £18,000–£30,999
- Level 3: £31,000–£51,999
- Level 4: >£52,000

**Townsend Deprivation Index**:

This index measures deprivation at the area level, considering unemployment, non-car ownership, non-home ownership, and household overcrowding. Scores were divided into quartiles, with a higher quartile indicating greater deprivation.

**Smoking Status**:

Participants were categorized based on their current smoking behavior:

- Never smoker
- Former smoker (having quit smoking at the time of assessment)
- Current smoker

**Alcohol Intake Frequency**:

Self-reported frequency of alcohol consumption was categorized as follows:

- Daily or almost daily
- 3–4 times per week
- 1–2 times per week
- Occasional (less than once a week)
- Never

**Hypertension Status**:

Hypertension was defined based on self-report or medical records, categorized as either present (Yes) or absent (No).

**Social Isolation and Loneliness**:

Social isolation was quantified using a composite score previously developed within the UK Biobank, based on living alone, frequency of engaging with friends or family, and participation in social activities. Loneliness was assessed through self-reported queries, identifying feelings of loneliness experienced over the past week.

**Depression**:

Depression was identified through self-reports, defined as experiencing a continuous state of depression lasting for an entire week.

**Dietary Scores**:

Dietary intake was quantified based on the frequency of consumption of fruits, vegetables, fish, and processed/red meat (inverted). Scores ranged from 0 to 20, with a higher score indicating a healthier diet. The scoring system was:

- Never = 0
- Less than once a week = 1
- Once a week = 2
- 2-4 times a week = 3
- 4-6 times a week = 4
- Once or more daily = 5

**Medication Use**:

Participants reported the use of any medication, categorized for this study as:

- Antihypertensive medications
- Blood-glucose-lowering medications
- Cholesterol-lowering medications

**APOE Allele Status**:

Determined through genotyping two single nucleotide polymorphisms (SNPs), rs7412 and rs429358. The APOE e4 allele status for each individual was categorized based on the combination of these SNPs into:

- No e4 alleles (e2/e2, e2/e3, or e3/e3 haplotypes)
- One e4 allele (e3/e4 and sporadic e2/e4 haplotypes)
- Two e4 alleles (e4/e4 haplotypes)

**Supplementary 2**. International Classification of Diseases (ICD) for the chronic diseases

Codes of International Classification of Diseases (ICD) were used to adjudicate HTN cases (ICD-10: I10, I11, I12, I13, I16), ASCVD cases (ICD-10: I25), CVD cases (ICD-10: I60, I61, I63, I64), ND cases (ICD-10: A81, F00, F01, F02, F03, F05, F10, G30, G31, I67, G20), MetS cases (ICD-10: E70, E71, E72, E73, E74, E75, E76, E77, E78, E79, E80, E83, E84, E85, E86, E87, E88, E89, E90) and T2DM cases (ICD-10: E11).

**Supplementary 3.** Formula for Odds Ratio

The odds ratio indicates how much likely participants with low or medium MVPA are to develop chronic diseases compared to those with high MVPA levels. The odds ratio is given as

$OR=\frac{ad}{bc}$,

where *a* = number of low or medium MVPA participants with chronic diseases, *b* = number of low or medium MVPA participants without chronic diseases, *c* = number of high MVPA participants with chronic diseases and *d* = number of high MVPA participants without chronic diseases.

**Supplementary 4**. HR Stratified by Age and BMI

*Age*

In evaluating the risk of developing chronic diseases across varying age groups, our analysis focused on the differences observed between middle-aged (45-60) and older (above 60) males and females over a 5-year period.

**Male Participants**:

Within middle aged cohort, we identified an elevated risk, with a 10-fold increase chance of developing hypertension compared to the younger age group (<45). Other chronic diseases did not show significant differences in risk within this time frame.

Among older males, the risk of developing certain chronic conditions showed a marked increase when compared to those under 45. Specifically, the likelihood of being diagnosed with hypertension was elevated by 20 times, the risk of ASCVD was 329% higher, the risk of metabolic syndrome was 246% greater. Risks for other chronic conditions did not demonstrate statistical significance.

**Female Participants**:

For females in the middle-aged group, the analysis did not reveal significant risks for the chronic diseases examined, indicating a comparable risk profile to the younger age group for the conditions studied.

In older females, significant risk increases were observed for hypertension and metabolic syndrome, with risks 467% and 426% higher, respectively, than in the younger age group. Like their male counterparts, the risks for other chronic diseases remained non-significant.

A very large non-significant HRs were observed for neurological diseases, including Cerebrovascular disease and neurodegenerative disease. This is primarily because the events of interest in these cases were rare, which can be resulted in large HRs.

*BMI*

We analysed the health risks associated with body mass index (BMI) among male and female individuals over a 5-year period, distinguishing between those classified as overweight (BMI = 25 to 30) and those classified as obese (BMI > 30).

**Male Participants**:

Within the overweight category, significant hazard ratios (HRs) were identified, indicating an elevated risk for type 2 diabetes (HR = 1.65, 95% CI: 1.16 to 2.36, p < 0.01) and hypertension (HR = 1.36, 95% CI: 1.19 to 1.56, p < 0.001).

Obese males exhibited even higher risks compared to their overweight counterparts. Notably, the risk for type 2 diabetes (HR = 4.55, 95% CI: 3.14 to 6.58, p < 0.001) and hypertension (HR = 2.08, 95% CI: 1.76 to 2.44, p < 0.001) were significantly elevated, alongside increases in ASCVD (HR = 1.37, 95% CI: 1.04 to 1.79, p < 0.05) and metabolic syndrome (HR = 1.35, 95% CI: 1.13 to 1.62, p < 0.01). The risk for cerebrovascular diseases did not significantly vary with BMI.

**Female Participants**:

Similarly, overweight females faced significant risks for type 2 diabetes (HR = 3.18, 95% CI: 2.13 to 4.76, p < 0.001), hypertension (HR = 1.30, 95% CI: 1.16 to 1.47, p < 0.001), and metabolic syndrome (HR = 1.15, 95% CI: 1.01 to 1.30, p < 0.05).

The obesity group showed a more pronounced risk across all examined conditions compared to the overweight group. This included remarkably high HRs for type 2 diabetes (HR = 8.50, 95% CI: 5.67 to 12.73, p < 0.001), hypertension (HR = 2.27, 95% CI: 1.98 to 2.61, p < 0.001), ASCVD (HR = 1.87, 95% CI: 1.35 to 2.59, p < 0.001), and metabolic syndrome (HR = 1.37, 95% CI: 1.17 to 1.62, p < 0.01).

**Supplementary Figures and Tables**

*Supplementary Figure Legends and File Names*

**Supplementary Figure S1:** Overview of an ML pipeline for accelerometer data processing.

**Supplementary Table S1:** Odds ratios, 95% confidence intervals, and p-values for chronic diseases by other covariates.

**Supplementary Table S2:** Hazard ratio of chronic disease development at 3 years with MVPA levels, gender, age, and BMI.
